# Hyperkalemia Induced Brugada Phenocopy: A Rare ECG Manifestation

**DOI:** 10.1155/2017/9464728

**Published:** 2017-02-23

**Authors:** Muhammad Ameen, Ghulam Akbar, Naeem Abbas, Ghazi Mirrani

**Affiliations:** ^1^Bronx-Lebanon Hospital Center, Department of Medicine, 1650 Selwyn Avenue, No. 10C, Bronx, NY 10457, USA; ^2^Lehigh Valley Hospital, Department of Cardiovascular Medicine, 1627 W. Chew St., Allentown, PA 18104, USA

## Abstract

Brugada syndrome (BrS) is an inherited disorder of cardiac ion channels characterized by peculiar ECG findings predisposing individuals to ventricular arrhythmias, syncope, and sudden cardiac death (SCD). Various electrolyte disturbances and ion channels blocking drugs could also provoke BrS ECG findings without genetic BrS. Clinical differentiation and recognition are essential for guiding the legitimate action. Hyperkalemia is well known to cause a wide variety of ECG manifestations. Severe hyperkalemia can even cause life threatening ventricular arrhythmias and cardiac conduction abnormalities. Most common ECG findings include peaked tall T waves with short PR interval and wide QRS complex. Since it is very commonly encountered disorder, physicians need to be aware of even its rare ECG manifestations, which include ST segment elevation and Brugada pattern ECG (BrP). We are adding a case to the limited literature about hyperkalemia induced reversible Brugada pattern ECG changes.

## 1. Introduction

Hyperkalemia is one of the most common electrolyte abnormalities that caused vast majority of ECG manifestation ranging from ST elevation to sine waves. It can also rarely cause Brugada pattern on ECG and it is very important for the clinicians to be aware of this presentation as treatment for both modalities is totally different, needing correction of potassium balance in hyperkalemia induced Brugada pattern and lot of investigations and ICD consideration in Brugada syndrome. Here we are presenting a case report of Brugada pattern on ECG caused by hyperkalemia that was reversed with the reversal of electrolyte imbalance.

## 2. Case Summary

A 43-year-old white male presented to ER with profound lower extremities weakness and severe muscle aches. Review of system was positive for decreased sensation in lower extremities. Past medical history included seizure disorder and splenectomy due to motor vehicle accident. He denied any history of syncope in the past. Family history was negative for sudden cardiac death or cardiac problem. He was homeless. He smoked 1 pack/day for 20 years and used cocaine and heroin. He was allergic to penicillin.

Physical examination showed vitals of blood pressure of 80/57 mmHg, heart rate of 88/minute, respiratory rate of 18/minute, oxygen saturation of 96% on 2 liters of supplemental oxygen through nasal cannula, and temperature of 97°F. He was awake and alert and well oriented to time and place. His power on both lower extremities was 0/5 with decreased touch sensation. There was no back tenderness or deformity. Anal tone was intact. Lungs were clear. Mucous membranes were dry. Heart exam showed regular normal first and second heart sounds with no murmur. Abdomen was benign. His lower extremities showed black gangrenous area on right calf that was swollen and tense.

**Figure 1:**
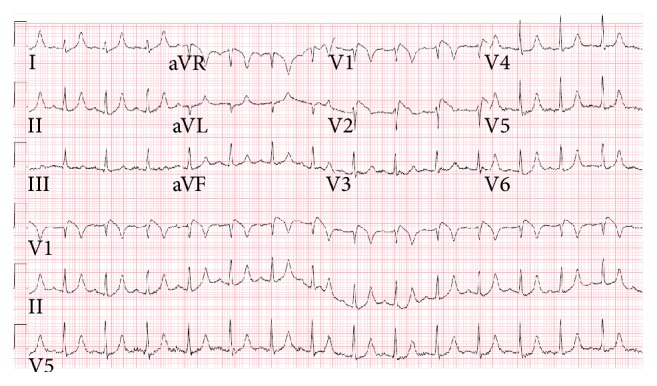
ECG on presentation showing ≥2 mm “coved type” ST elevation and a T-wave inversion in precordial leads V1 and V2 (type 1 Brugada pattern). Axis is normal and QRS are not widened.

His laboratory data showed potassium of 8.2 mEq/L (3.5–5 mEq/L) on nonhemolysed sample. Other laboratory data were as follows: Blood Urea Nitrogen (BUN) 40 mg/dL (8–26 mg/dL), creatinine 4 mg/dL (0.5–1.5 mg/dL), sodium 135 mEq/L (135–145 mEq/L), chloride 98 mEq/L (98–108 mEq/L), serum bicarbonate 13 mEq/L (24–30 mEq/L), magnesium 2.2 mEq/L, hemoglobin 16.7 g/dL (12–17 G/dL), white count 23,000/mm^3^ (4800–11,000/mm^3^), platelet count 308,000/mm^3^ (150,000–400,000/mm^3^), INR 1.3, CPK 46,6879 units/L, CK-MB 0.377 ng/mL (<5 ng/mL), troponin 0.66 (<1 ng/mL), AST 1630 U/L (9–48 U/L), ALT 372 U/L (5–40 U/L), alkaline phosphatase 100 U/L (56–119 U/L), total bilirubin 0.2 mg/dL (0.2–1.2 mg/dL), and GGT 7 U/L (8–54 U/L). Urinalysis was significant for +3 blood with 5–9 RBC and +2 proteinuria; urine specific gravity was 1.009. His urine was positive for myoglobin. His chest X-ray was clear. MRI thoracolumbar spine did not show any spinal cord abnormality. His EKG was consistent with type 1 Brugada pattern on presentation (Figure 1). Transthoracic echocardiography (TTE) showed normal systolic and diastolic function with no valvular abnormality.

## 3. Hospital Course

**Figure 2:**
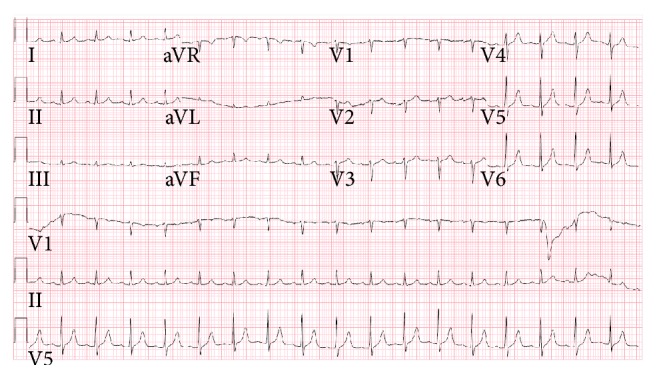
Showing resolution of Brugada pattern ECG changes after hyperkalemia was resolved.

43-year-old male presented with profound leg weakness, acute renal failure, severe hyperkalemia, rhabdomyolysis, and compartment syndrome. He was severely dehydrated. His hyperkalemia was treated with calcium gluconate, insulin, glucose, and sodium polystyrene sulfonate. He was aggressively hydrated with bicarbonate solution. Emergent fasciotomy was done for his compartment syndrome. His leg weakness was attributed to severe hyperkalemia that was resolved later on along the course of his hospital stay. MRI thoracolumbar spine did not show any spinal cord abnormality. His electrocardiogram showed type 1 Brugada pattern induced by severe hyperkalemia and it was resolved after potassium became normal (Figure 2). Intermittent hemodialysis (IHD) was initiated due to anuric acute renal failure and after 8 sessions of IHD, his kidney functions recovered. His hospital course was complicated by MSSA bacteremia, which was adequately treated with antibiotics. He was transferred to rehabilitation facility in a stable condition.

## 4. Discussion

First introduced in 1992, BrS is a hereditary disorder (autosomal dominant with variable expression) associated with syncope, life threatening arrhythmias leading to sudden cardiac death [1]. It is defined as characteristic electrocardiographic (ECG) changes associated with one or more of the following clinical findings: documented ventricular fibrillation (VF), polymorphic ventricular tachycardia (VT), family history of sudden cardiac death at age younger than 45 years, family history of type 1 Brugada pattern ECG changes, inducible VT during electrophysiology study, and unexplained syncope suggestive of a tachyarrhythmia or nocturnal agonal respiration [2].

Three characteristic ECG patterns have been described in BrS. Type 1 ECG findings include spontaneous or drug induced (intravenous Class I antiarrhythmic drugs) ST segment elevation (≥2 mm) with an upward convexity “coved type” and a T-wave inversion. Type 2 Brugada pattern has ≥2 mm ST elevation with “saddle back” morphology, a trough ≥1 mm ST elevation, and then either a positive or biphasic T wave. Type 3 Brugada pattern has either coved type 1 or type 2 saddle back configuration but ST elevation is <1 mm. Interestingly, presence of type 2 and type 3 ECG patterns is not diagnostic of BrS. It requires reproduction of type 1 ECG pattern on drug provocative test to confirm BrS. All 3 types are characterized by ECG changes limited to the right precordial leads, V1 to V3, positioned in the 2nd, 3rd, or 4th intercostal space. Axis is usually normal and QRS complex is narrow or mildly widened [3].

Clinical manifestations associated with BrS range from chest pain, syncope, to SCD (more often at night, responsible for 4% of all SCD). Actual data on the true prevalence of BrS is not available. However, it is more prevalent in Asian and Southeast Asian populations such as Thailand, Philippines, and Japan as high as 0.5–1 per 1000. Males have 8–10 times higher chance of having BrS as compared with females [2, 3].

BrS has traditionally been reported as an electrical disorder in the absence of structural heart disease. Recent research documents reveal some evidence of structural or pathological changes especially of the right ventricle. Most recently Gray et al. have raised an interesting concept of BrS as being a heterogeneous disorder with common ECG changes with or without structural heart defect. Structural heart abnormalities include subtle morphological changes in the right ventricular such as dilated right ventricular outflow tract (RVOT) or histological finding of fibrofatty infiltration or inflammation of RVOT [4].

So far 12 genes have been reported to be involved in encoding for sodium, calcium, or potassium channels. The most common one is a loss of function mutation in the gene* SCN5A, *encoding for alpha subunit of cardiac sodium channels. Brugada phenotype has been associated with genes involving either decrease in the inward sodium or calcium current or increase in outward potassium current. More broadly we can define BrS as genetic disorder of channelopathy with a combination of functional defects in the repolarization and/or depolarization phase and localized structural abnormality leading to common phenotype [5].

Brugada pattern (BrP) ECG or Brugada sign is termed when characteristics ECG findings of BrS are observed in an asymptomatic patient with no other criteria. Our case demonstrated reversible ECG features and did not have any of the characteristics in the criteria. With correction of hyperkalemia, spontaneous resolution of Brugada sign was noted. So, we did not proceed with electrophysiological study.

Recognition of BrS from BrP is crucial as BrS has an increased risk of SCD and may require treatment. An implantable cardioverter defibrillator (ICD) has shown clear evidence in preventing life threatening arrhythmias and SCD. The 2013 expert consensus guidelines by HRS/EHRA/APHRS strongly recommend ICD implantation in patients with aborted SCD and/or sustained VT with or without syncope. Pharmacological treatment like quinidine can be used in patients in whom ICD is indicated but cannot be implanted (patient refusal or contraindication), arrhythmic storms (more than 2 episodes of VT/VF in 24 hours) and history of sustained supraventricular arrhythmias. Anecdotal case reports have documented benefit of radiofrequency catheter ablation but further studies are needed to prove its long-term substantial efficacy. Family history of SCD or drug provoked BrP or asymptomatic BrS is not an indication for ICD implantation as risk of critical events is very low. Currently, the roles of electrophysiological study or a pertinent family history of SCD remains debatable [3].

BrP ECG is a rare but challenging finding. It draws physician's attention to rule out BrS as a possible diagnosis. There are many conditions that can induce BrP ECG, sometimes making it difficult to separate it from genetic BrS. Reversible BrP ECG changes have been reported to occur due to electrolyte disturbances like hyperkalemia, hyponatremia, acidosis, hyperglycemia, cocaine, alcohol, drugs like neuroleptics or tricyclic antidepressants, type 1C antiarrhythmic, fever, and electrocution [6–10].

Hyperkalemia is a very common and potentially life threatening electrolyte disorder. It is associated with wide variety of ECG changes ranging from minimal ECG changes to arrhythmias and sine wave pattern [11]. Rare ECG manifestations of hyperkalemia include ST elevation and reversible BrP [12, 13]; since data is limited to case reports only, exact electrophysiological mechanism is uncertain. It is predicted that with increasing level of potassium concentration, progressive ECG changes will be observed but this relationship sometimes is not evident [14] (Table 1). Severe hyperkalemia, in rare circumstances, can be associated with minimal ECG findings lacking characteristic hyperkalemia related features or even normal ECG. Hyperkalemia related ECG changes are more likely to be severe and progressive in the setting of concurrent occurrence of metabolic acidosis, hypocalcemia, and hyponatremia. Conversely, metabolic alkalosis, hypercalcemia, and hypernatremia can mask the effects of hyperkalemia on membrane potential minimizing typical ECG changes [15–17].

Presumably severe hyperkalemia inactivates the cardiac sodium ion channels by decreasing the resting membrane potential. It has been observed mainly in critically ill male population. Widened QRS complex and absent P waves sometimes seen in hyperkalemia induced BrP can help distinguish it from genetic BrS [18]. Our patient did not reveal widened QRS or absent P wave making it even more difficult to differentiate but he did not meet the other criteria for genetic BrS. To our knowledge, only 27 case reports have been reported where hyperkalemia related BrP was described.

## 5. Conclusion

Brugada pattern ECG changes can occur in the setting of hyperkalemia, and these changes are reversible upon correction of this common electrolyte abnormality.

Prompt recognition and early treatment of hyperkalemia can be lifesaving. They can completely reverse these changes and avoid the unnecessary interventions. Physicians should be mindful of this rare presentation of a very common electrolyte disorder.

## Figures and Tables

**Table 1:** ECG changes of hyperkalemia depending upon severity with possible mechanism.

Hyperkalemia	Expected ECG finding	Possible mechanism
Mild (5.5–6.5 mEq/L)	Tall, peaked T waves in precordial leads or pseudonormalization (i.e., upright) of already inverted T waves such as in LVH	Acceleration of terminal phase of repolarization

Moderate (6.5–8.0 MEQ/L)	Tall T waves Prolonged PR interval Short QT interval Flattening of P waves Wide QRS complex	Inactivation of cardiac sodium channels due to decreased atrial and myocardial transmembrane potential resulting in reduced action potential Atrial tissue is more sensitive so atrial changes in P wave and PR interval are seen earlier than ventricular changes in QRS complex

Severe (>8.0 MEQ/L)	Absent P waves Conduction defects such as fascicular blocks, bundle branch blocks, complete heart block Progressively wide QRS complex Escape beats or rhythm Eventually “sine wave” morphology VT, VT, asystole	Suppressed or delayed conduction of SA and AV nodal impulse

LVH: left ventricular hypertrophy; SA: sinoatrial; AV: atrioventricular.

## Abbreviations

LVH: Left ventricular hypertrophy
SA: Sinoatrial
AV: Atrioventricular
BrS: Brugada syndrome
BrP: Brugada pattern EKG changes
VT: Ventricular tachycardia
VF: Ventricular fibrillation
IHD: Intermittent hemodialysis
ECG: Electrocardiogram.
